# Abcès idiopathique de la thyroïde: à propos d'un cas colligé au Centre Marc Sankale Dakar

**DOI:** 10.11604/pamj.2019.34.120.19963

**Published:** 2019-10-30

**Authors:** Diallo Ibrahima Mané, Diédhiou Demba, Sow Djiby, Ndour Michel Assane, Gueye Adja Nafissa, Barrage Ahmet Limane, Thioye El Hadji Mamadou Moussa, Ka-Cissé Marie, Sarr Anna, Ndour Mbaye Maimouna

**Affiliations:** 1 Clinique Médicale II, Centre Hospitalier Abass Ndao, Université Cheikh-Anta-Diop, Dakar, Sénégal

**Keywords:** Abcès, thyroïde, Sénégal, Abscess, thyroid, Senegal

## Abstract

L'abcès de la thyroïde est une maladie très rare en raison de sa situation anatomique et de la physiologie de la glande. Il survient la plus part du temps sur un terrain d'immunodépression. Le diagnostic est facile mais on y pense pas souvent et repose sur l'échographie. L'examen bactériologique permet de retrouver le germe responsable. Nous rapportons le cas d'un patient mauritanien vivant dans ledit pays et qui avait consulté pour une tuméfaction cervicale antérieur douloureuse associée à une fièvre. Le diagnostic était posé à l'échographie et confirmé par l'examen anatomopathologique du liquide de ponction, sans étiologie retrouvée à la bactériologie. L'évolution était marqué par une régression des symptômes sous antibiothérapie, avec une normalisation de la structure de la glande après 2 mois.

## Abstract (en)

Thyroid abscess is a very rare disease due to its anatomical position and the physiology of the gland. It most often occurs in the immunosuppressed population. The diagnosis is easy and is based on ultrasound. However it is often unrecognised. Bacteriological examination allows to identify the germ responsible for this condition. We report the case of a Mauritanian patient living in the said country presenting with painful cervical swelling associated with fever. The diagnosis was based on ultrasound and confirmed by fluid examination. Bacteriological assessment didn't identify the cause of this condition. Patient's outcome was marked by regression of symptoms under antibiotic therapy, with normalization of the gland structure after two months.

## Introduction

L'abcès est une collection purulente au sein d'une cavité néoformée. La location à la thyroïde, est une entité rare (0,1%) [1] et inhabituelle du fait de son anatomie qui le protège des infections. De façon générale, les organes résistants aux infections, semblent être exposés en cas d'immunodépression. L'abcès surviennent généralement sur une glande morphologiquement anormale: acquise (goitre, nodule), anomalie congénitale (fistule de la 4^ème^ fente), ou iatrogène (cytoponction) [2].

## Patient et observation

**Figure 1:**
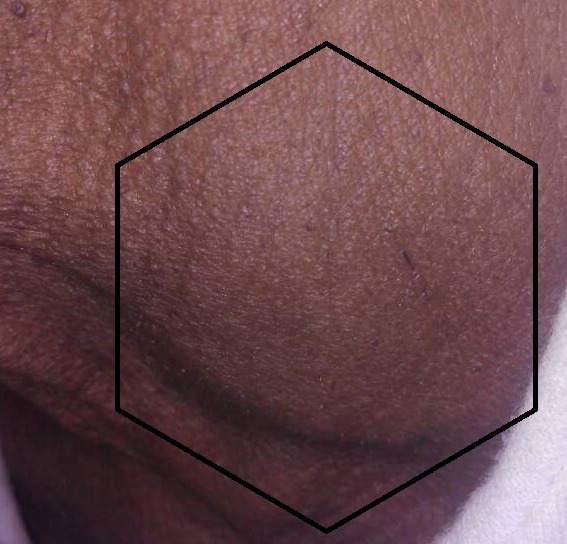
Abcès de la thyroïde vue de profil centre

**Figure 2:**
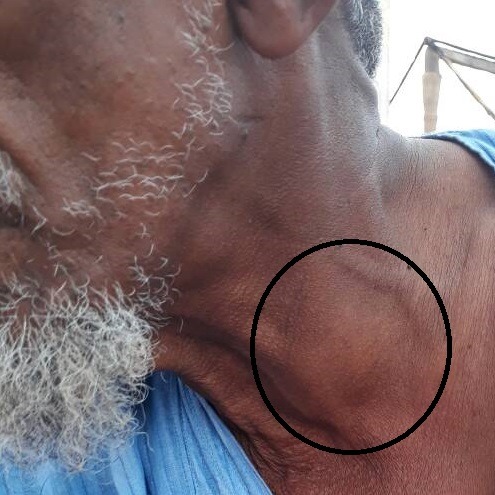
Abcès de la thyroïde vue générale de face

Nous rapportons un cas rare d'abcès thyroïdien, survenu chez un patient mauritanien de 67 ans avec comme terrain d'immunodépression l'âge, seul l'hypertension artérielle était la comorbidité associée. Le patient s'est présenté en consultation d'urgence le 24/08/2017 pour une tuméfaction cervicale antérieure gauche (Figure 1, Figure 2), douloureuse, fébrile évoluant depuis 21 jours. Les premiers symptômes étaient: une gêne à la déglutition, une fièvre non objectivée et une douleur à la palpation du cou (région thyroïdienne). La symptomatologie s'est aggravée au fil des jours, avec l'apparition d'une tuméfaction cervicale antérieure augmentant progressivement de volume, associée à une dysphagie, une dysphonie, dans un contexte d'altération de l'état général et de fièvre vespéro-nocturne.

L'examen physique avait objectivé une masse cervicale antérieure, arrondie, basi cervicale médiane déviée à gauche, mesurant 6 cm de grand axe (Figure 1, Figure 2). La masse était tendue et rénitente, peu douloureuse à la palpation, avec une peau en regard qui était saine. L'examen n'avait pas retrouvé d'adénopathies superficielles ni profondes. La masse était mobile à la déglutition, associée à une dysphonie, une fièvre à 38°C avec des sueurs sans frissons, le patient n'était pas dyspnéique. Le bilan biologique du 17/08/17 avait montré: une hyperleucocytose à prédominance neutrophile à 13,92x10^6^ globules blancs et à l'électrocardiogramme on avait un infarctus antéro-septal. Le bilan du 24/08/17 avait montré: hyperleucocytose à prédominances neutrophile à 16,7x10^6^ globules blancs, une C Réactive Protéine (CRP) à 96 mg/l, la thyréostimuline ultrasensible (TSH-US) normale à 0,27 ui/l. L'échographie du 18/08/2017 montrait: un nodule multiloculaire du lobe gauche de la thyroïde bien limite sans adénopathie satellite. L'échographie du 24/08/2017 montrait: un nodule du lobe gauche de la thyroïde arrondie, bien limite, avasculaire, massivement nécrosé, sans adénopathie satellite, ni de calcification d'environ 67mm/46mm. L'indication d'une ponction guidée par l'échographie était posée et avait ramené un liquide épais purulent jaune bien lié non fétide. L'examen cytologique montre la présence de plusieurs polynucléaires majoritairement altères, associé à des lymphocytes et plasmocytes, sans cellule suspect de malignité. A la bactériologie, l'examen à l'état frais et après culture ne retrouve aucun germe y compris la recherche de Bacille de Koch en culture. Le diagnostic d'abcès de la thyroïde idiopathique a été retenu et le malade mis sous traitement. Les autres bilans retrouve à l'électrocardiogramme un infarctus antéro-septal et à l'échographie une hypertrophie concentrique des parois avec une fraction d'éjection conservée à 75% évoquant une cardiomyopathie ischémique.

L'évolution était favorable sous bi-antibiothérapie première à base d'amoxicilline et acide clavulanique 2 g/jour et antalgique pallier II. Elle était marquée par une régression de la masse cervicale et une atténuation de la douleur. Une prise en charge de son infarctus antéro-septal a été prise en compte et le malade mis sous nebivelol 5 mg et acide acétyle salicylique. La normalisation des paramètres biologiques a été obtenue après 5 jours de traitement antibiotique avec persistance à l'échographie d'un macronodule. Le traitement antibiotique a été arrêté après 15 jours et la restitution ad integrum de la glande était obtenue après le contrôle du 2^ème^ mois.

## Discussion

L'abcès de la thyroïde est une pathologie rare (0,1%) plus fréquente chez les enfants en raison des malformations pouvant survenir au cours du développement embryonnaire [3]. Une large étude de Yu *et al.* [4], avait noté une répartition égale entre les deux sexes. L'abcès de la thyroïde peut survenir à tout âge. Dans cette même étude il avait été démontré que cette pathologie pouvait toucher une large population. Chez l'adulte, de multiples étiologies sont en cause. L'infection peut résulter soit par action directe: inoculation iatrogène d'un corps étranger, tels que la cytoponction thyroïdienne, une perforation de l’œsophage ou de l'hypo pharynx, soit indirecte par dissémination hématogène ou lymphatique à partir d'un foyer à distance. D'autres facteurs induisant l'infection de la thyroïde comprennent: une modification de la morphologie thyroïdienne chez l'enfant, immunodépression chez l'adulte [5]. Chez notre patient, le seul élément d'immunodépression retrouve était l'âge avancé. Le tableau clinique inflammatoire était incomplet chez notre patient, ceci s'expliquait probablement par l'utilisation des anti-inflammatoires avant consultation. Yu et ses collaborateurs [4] avaient étudiés 191 cas de 1980 à avril 1997 et les ont comparés à 224 cas (1900-1980). Ils avaient découvert que le nombre de patients immunodéprimés augmentaient, concomitamment avec le nombre de cas de thyroïdite suppurée. Ce qui était important à retenir est que la thyroïdite à germe banal était associée à un état euthyroïdien (83,1%), alors que pour les germes spécifiques mycobactéries on avait des hyperthyroïdies (50%) et des hypothyroïdies (62,5%) pour les mycoses [6].

Un diagnostic précoce est important car la progression est rapide et pourrait être fatale. L'échographie est la technique d'imagerie de référence dans les pays en voie de développement pour le diagnostic des maladies de la thyroïde [2]. Elle peut également être utilisée pour la ponction écho-guide. Au Maroc en 2012, sur un cas d'abcès de la thyroïde, la cytoponction et l'échographie ont permis de poser le diagnostic [2]. Sauf autre indication, il n'est pas nécessaire de faire recours à un scanner ou une imagerie par résonance magnétique pour le diagnostic d'un abcès de la thyroïde [7]. Les étiologies des abcès de la thyroïde sont multiples, les infections à streptococcies et staphylococcus sont très fréquentes et représentent 70% des cas [8]. Mais, aussi d'autres germes ont été rapportés dans la littérature. Les mycobactéries bien que rare, existent et sont rapportées dans la littérature. Leur localisation dans la thyroïde représentent 0,1 à 0,4% de toutes les localisations de la tuberculose et sont en général multiple simulant un goitre multi nodulaire [9]. Dans notre cas aucun germe n'a été trouvé définissant ainsi le terme d'abcès de la thyroïde idiopathique. Les antimicrobiens et le drainage chirurgical de l'abcès constituent le traitement de choix. Dans les cas de fistules sinus pyriformes, des techniques de chimio cautérisation sont utilisées, car moins invasives. Dans notre cas, une décision d'un traitement médical était adoptée avec une surveillance des risques de complication a type de fistulisation médiastinale, et trachéal. L'évolution dans notre cas par une amélioration des paramètres clinique après 72 heures, des paramètres biologiques après une semaine.

## Conclusion

L'abcès suppure de la thyroïde est décrit comme une entité rare et survenant dans un contexte particulier d'immunodépression. Le diagnostic est souvent posé à l'échographie et la cytoponction. Devant syndrome infectieux associé à une image unique d'abcès un germe banal est incriminé, et en cas d'existence de plusieurs nodules une tuberculose doit être suspectée jusqu'à preuve du contraire, mais l'étiologie est rarement retrouvée. Les lésions infectieuses de la thyroïde répondent bien au traitement antibiotique avec ou sans chirurgie. Vu tôt, l'évolution est favorable et ne nécessite pas de chirurgie.

## Conflits d’intérêts

Les auteurs ne déclarent aucun conflit d'intérêts.
